# 3 months vs 12 months of romosozumab for postmenopausal osteoporosis (LIDA): an open-label, non-inferiority, randomised controlled trial

**DOI:** 10.1016/S2213-8587(25)00319-5

**Published:** 2026-01-29

**Authors:** Benjamin Z Leder, Sabashini K Ramchand, Mackenzie Jordan, Savannah Ryan, Aparna Patnaik, Hang Lee, Joy N Tsai

**Affiliations:** Department of Medicine, Harvard Medical School, Endocrine Unit, Massachusetts General Hospital, Boston, MA USA; Department of Medicine, Lerner College of Medicine, Case Western Reserve University, Endocrine Unit, Cleveland Clinic, Cleveland, OH, USA; Department of Medicine, Harvard Medical School, Endocrine Unit, Massachusetts General Hospital, Boston, MA USA; Department of Medicine, Harvard Medical School, Endocrine Unit, Massachusetts General Hospital, Boston, MA USA; Department of Medicine, Harvard Medical School, Endocrine Unit, Massachusetts General Hospital, Boston, MA USA; Massachusetts General Hospital, Biostatistics Center, Boston, MA, USA; Department of Medicine, Harvard Medical School, Endocrine Unit, Massachusetts General Hospital, Boston, MA USA

## Abstract

**Background:**

Postmenopausal osteoporosis is a highly prevalent disease associated with substantial morbidity and mortality. The most recently introduced osteoporosis medication is romosozumab, a monoclonal antibody with a unique mechanism of action that increases bone mineral density (BMD) more than other agents by both stimulating new bone formation and inhibiting resorption. The drug’s stimulation of bone formation, however, wanes after several months. The use of romosozumab is limited by cost, the inconvenience of monthly clinic-administered injections, and its cardiovascular risk profile. In this trial, we aimed to test the hypothesis that a shorter course of romosozumab might be equally effective as the standard regimen.

**Methods:**

We did a 12-month, prospective, open-label, randomised, controlled, non-inferiority trial of 50 postmenopausal women at high risk of fracture. The study was done at a single academic medical centre in the USA. Participants were randomly assigned to receive 3 months of romosozumab (210 mg by subcutaneous injection, monthly) followed by 9 months of denosumab (60 mg by subcutaneous injection, every 6 months; 3-month ROMO group) or 12 months of romosozumab (12-month ROMO group). The primary endpoint was the percentage change in total hip BMD. The non-inferiority threshold was set at 2%. The trial was registered at ClinicalTrials.gov, NCT05010590.

**Findings:**

Between March 2, 2022, and May 2, 2023, we screened 188 participants. Of those, 102 (54%) were ineligible and 36 (19%) declined to participate. We randomly assigned 50 participants to either the 3-month ROMO group (24 [48%] participants) or the 12-month ROMO group (26 [52%] participants). Study participants completing at least one post-baseline visit were included in the analysis (modified intention-to-treat analysis). The mean age of participants was 69·6 years (SD 4·5). The mean 12-month change in total hip BMD was 5·7% (SD 3·3) in the 3-month romosozumab group and 6·0% (3·2) in the 12-month romosozumab group, meeting the prespecified non-inferiority threshold. Adverse events (back pain, cough, fatigue, headache, joint pain, muscle cramps, muscle pain, palpitations, paraesthesia, reaction at injection site, rhinorrhoea, skin rash, and swelling) were balanced between groups.

**Interpretation:**

In postmenopausal women at high risk of fracture, 3 months of romosozumab followed by 9 months of denosumab was non-inferior to 12 months of romosozumab in increasing total hip BMD. Given the expense, injection burden, and potential adverse effects of romosozumab, this abbreviated approach could broaden access to this uniquely effective therapy.

**Funding:**

US National Institute of Arthritis and Musculoskeletal and Skin Diseases and US National Center for Advancing Translational Science.

## Introduction

Postmenopausal osteoporosis is a major public health concern that is associated with substantial morbidity, mortality, and health-care expenditure.^1^ The prevalence of osteoporotic fracture differs by country but it has been estimated that worldwide, approximately 40–50% of all postmenopausal women will experience a fragility fracture during their lifetime.^2^ Although many types of fragility fractures can have severe consequences, hip fractures are particularly devastating as they lead to admission to long-term care facility in 20% of cases and a doubling of 1-year mortality.^3,4^

Osteoporosis medications reduce vertebral and non-vertebral fracture risk by either inhibiting bone resorption (antiresorptive agents) or stimulating bone formation (osteoanabolic agents).^5^ Romosozumab, a US Food and Drug Administration-approved monoclonal antibody that binds and inhibits sclerostin, has both osteoanabolic and antiresorptive properties, stimulating osteoblastic bone formation while inhibiting osteoclastic activity. In clinical trials, a 12-month course of romosozumab followed by 1–2 years of antiresorptive therapy has been shown to increase bone mineral density (BMD) more than any monotherapy and to reduce fracture risk more effectively than the most commonly used osteoporosis therapy, alendronate.^6,7^

Importantly, however, although romosozumab both stimulates bone formation and inhibits resorption, the drug’s osteoanabolic effects are transient, with maximal gains in bone formation markers observed within days and reverting to baseline after 2–3 months of treatment.^8,9^ Conversely, romosozumab’s antiresorptive effects, which are modest, are sustained over the entire year-long treatment course. This rapid waning in bone formation after several months of exposure raises questions about the necessity and utility of a full 12-month regimen, especially because romosozumab’s antiresorptive properties are relatively modest when compared with other antiresorptive agents such as denosumab or bisphosphonates.^6,10^

Concerns about romosozumab’s cardiovascular safety have also emerged. Data from published clinical trials concerning the incidence of major adverse cardiovascular events (MACE: defined as myocardial infarction, stroke, and cardiovascular death) are conflicting. A phase 3 trial comparing romosozumab with alendronate in over 4000 postmenopausal women reported a significant increase in MACE (2·5% *vs* 1·9%), whereas a separate phase 3 placebo-controlled trial in over 7000 postmenopausal women reported no significant difference.^6,7^ A numerically (but not statistically significant) higher incidence of MACE was reported in a placebo controlled study of 245 men (4·9% in the romosozumab group *vs* 2·5% in the placebo group).^11^ These findings have led to a boxed warning for romosozumab in the USA and prominent warnings on the labels of all countries where it is approved for use. The use of romosozumab is further constrained by practical challenges such as its high cost, the need for monthly clinic-administered injections, and insurance-imposed restrictions—all of which are associated with reduced treatment initiation and high discontinuation rates.^12^ In real-world settings, adherence to the standard 12-month regimen is poor, with discontinuation rates in the USA reported to exceed 50% by the end of the treatment course.^13^ Data on adherence from other countries are limited, although a study in Taiwan reported only a slightly better adherence rate of 64%.^14^ Given that the osteoanabolic effects of romosozumab appear to be concentrated within the initial months of treatment, we hypothesised that a shorter course of therapy might preserve efficacy while improving affordability, adherence, and safety.

## Methods

### Study design and participants

We did a 12-month, prospective, open-label, randomised, controlled, non-inferiority trial designed to test the hypothesis that in postmenopausal women at high fracture risk short-term administration of romosozumab followed by denosumab would result in non-inferior increases in total hip areal BMD compared with the standard 12-month romosozumab regimen. The trial was done at Massachusetts General Hospital in Boston, MA, USA. All participants provided written informed consent, and the protocol was approved by the Mass General Brigham Institutional Review Board. The study was supported by the National Institute of Arthritis and Musculoskeletal and Skin Diseases (NIAMS) and monitored by a NIAMS-appointed Data Safety Monitoring Board.

We enrolled postmenopausal women aged 45 years or older through referrals from specialty clinics and targeted mailings (age >60 years) within the Boston area. The inclusion criteria were developed to conform with the US Food and Drug Administration-approved indications for romosozumab and denosumab. All women were at high risk of fracture defined as either previous history of fragility fracture or a BMD T score of −2·5 or less at the total hip, femoral neck, or lumbar spine. Key exclusion criteria were use of oral bisphosphonates or denosumab within 12 months; use of oestrogens, selective oestrogen receptor modulators, or calcitonin within 3 months; more than 14 days of glucocorticoid exposure within 6 months; use of teriparatide, abaloparatide, or intravenous bisphosphonates within 3 years; or any previous use of romosozumab or strontium. Other exclusion criteria were serum alkaline phosphatase greater than two times the upper normal limit, stage 4 or stage 5 chronic kidney disease (glomerular filtration rate <30 mL/min per 1·73 m^2^), hypercalcaemia or hypocalcaemia, elevated blood parathyroid concentration, serum 25-hydroxyvitamin D less than 20 ng/mL, haematocrit less than 32%, history of malignancy (except basal cell carcinoma), history of myocardial infarction or stroke within the preceding year, history of unstable angina or transient ischaemic attack in the past year, current atrial fibrillation, uncontrolled eczema, congenital or acquired bone disease other than osteoporosis, history of osteonecrosis of the jaw, extensive dental work involving extraction or dental implants in the past 3 months or planned in the upcoming 12 months, or any health condition that, in the opinion of the study physician, significantly increases the risk of cardiovascular events.

### Randomisation and masking

Participants were randomly assigned (1:1) to receive either 3 months of romosozumab (210 mg subcutaneously, monthly) followed by 9 months of denosumab (60 mg subcutaneously at month 3 and month 9; 3-month ROMO group) or 12 months of romosozumab (210 mg subcutaneously, monthly; 12-month ROMO group; appendix p 3). Randomisation was computer-generated with a randomly varying block size. Participants were stratified by age (<65 years or ≥65 years) and previous oral bisphosphonate use (yes or no). The study statistician generated the sequence at the initiation of the trial.

### Procedures

Assessments were made at each study visit (months 0, 3, 6, and 12). Participants reporting dietary calcium intake below 1200 mg/day received calcium supplementation to achieve at least 1200 mg/day and vitamin D3 (400 IU/day); others received vitamin D3 only.

### Outcomes

The pre-determined primary endpoint of the study was the 12-month change in total hip areal BMD assessed by dual-energy x-ray absorptiometry. Secondary endpoints were BMD of the femoral neck and lumbar spine. Exploratory endpoints were serum markers of bone turnover and BMD of the distal radius. Safety and adverse events were assessed by study investigators at each study visit.

BMD of the total hip, femoral neck, lumbar spine, and distal radius were assessed using the same dual-energy x-ray absorptiometer (Horizon, Hologic, Marlborough, MA) at the Massachusetts General Hospital Bone Density Center with coefficients of variation of 0·006 g/cm^2^ for total hip, 0·009 g/cm^2^ for femoral neck, and 0·005 g/cm^2^ for lumbar spine. Serum markers of bone turnover—β-isomer of C-terminal telopeptide of type I collagen (CTX) and procollagen type I N-terminal propeptide (P1NP)—were measured in morning fasting samples and assayed in batches at study completion by electrochemiluminescence (iSYS, Immunodiagnostic Systems, Scottsdale, AZ, USA). The inter-assay coefficients of variation were 6·0% for CTX and 5·0% for P1NP, and intra-assay coefficients of variation were 3·2% for CTX and 2·9% for P1NP. For CTX values below the limit of assay detection (<0·023 ng/mL), a value of 0·023 ng/mL was imputed. The individuals assessing all endpoints were blinded to group assignment.

### Statistical analysis

Analyses were done in the modified intention-to-treat population, which included all participants who received the study medications and completed at least one post-baseline visit. Baseline demographic and clinical characteristics were summarised using descriptive statistics. Non-inferiority testing for the primary endpoint was assessed by determining if the 90% CI of the difference in the 12-month change resided within ±M, where M is the prespecified non-inferiority margin. M was derived by calculation of both the fixed margin method, which uses a value ranging from 50% to 75% of the difference between the values found in control and placebo (method 1), and the US Food and Drug Administration-advised method, which uses 50% of the lower limit of the 95% CI of the difference between the values found in the control and in the placebo groups (method 2). Our calculations were based on the phase 3, placebo-controlled trial of romosozumab.^6^ For the primary endpoint of total hip BMD, using method 1, the M value would be between 1·8% and 3·5% and, using method 2, the M value would be 2·8% (lower CI 5·6%). We set the non-inferiority margin (M) at a conservative value of 2%, requiring a sample size of 18 trial participants per group to achieve 90% power at a 5% type-1 error rate. Importantly, this statistical inferiority threshold is also similar to what is currently accepted as clinically relevant. Specifically, in an analysis derived from individual patient data from 16 randomised controlled trials and over 60 000 patients, total hip BMD gains of 1·83% or greater (*vs* placebo) were associated with a significant reduction in all fractures.^15^ Between-group differences in other variables with multiple timepoints were examined using longitudinal random-intercept linear mixed effects models. A p value of 0·05 or less was considered statistically significant for all secondary and exploratory outcomes.

### Role of the funding source

The funder of the study had no role in study design, data collection, data analysis, data interpretation, or writing of the report.

## Results

**Figure 1:**
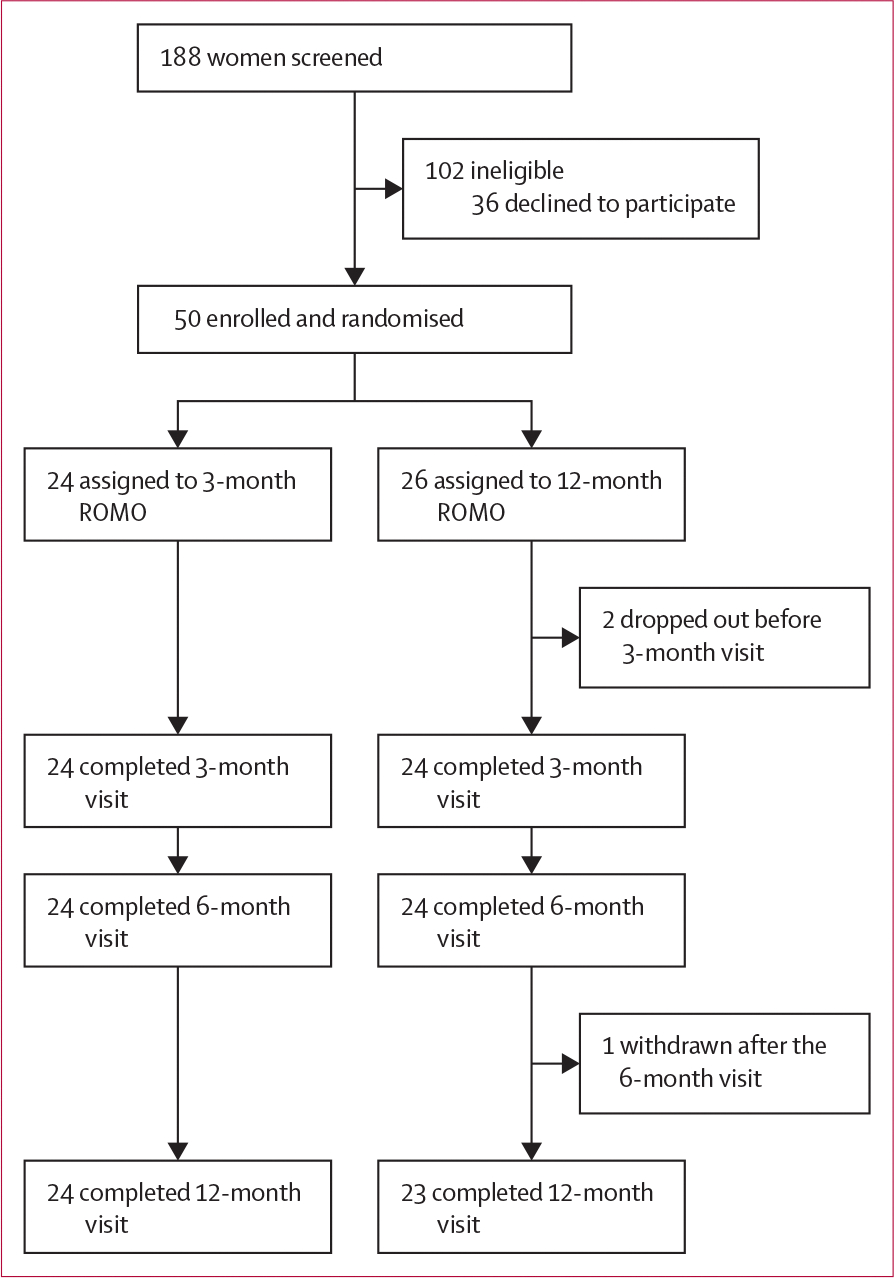
Trial profile 3-month ROMO=3 months of romosozumab followed by 9 months of denosumab. 12-month ROMO=12 months of romosozumab.

Between March 2, 2022, and May 2, 2023, we screened 188 participants (figure 1). Of those, 102 (54%) were ineligible (98 [96%] had BMD above threshold, one with a history of bisphosphonate related subtrochanteric fracture, one with upcoming oral surgery, and two who used exclusionary medications), and 36 (19%) declined to participate. We randomly assigned 50 participants to either the 3-month ROMO group (24 [48%] participants) or the 12-month ROMO group (26 [52%] participants). The mean age of participants was 69·6 years (SD 4·5). 24 participants in each group completed at least one post baseline visit and were included in the analysis. There were no statistically significant differences between groups in terms of baseline characteristics (table 1).

**Figure 2:**
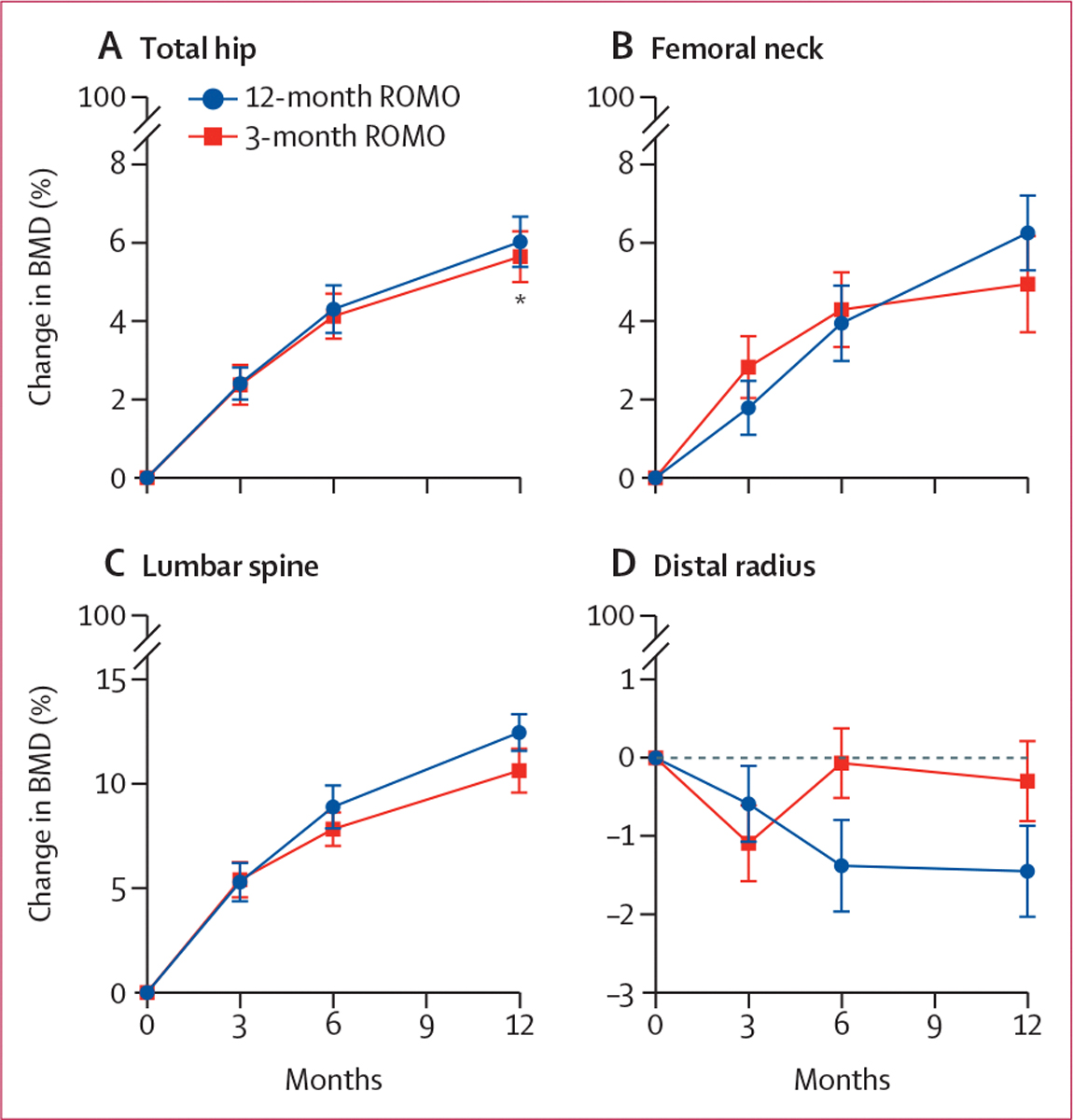
Change in BMD over the 12-month treatment period Figure shows the mean percent change from baseline in BMD at the labelled anatomic sites in the two treatment groups. Error bars indicate the SE of the mean. Asterisks indicate that the difference meets the non-inferiority threshold. No statistically significant between-group differences were found at any timepoint at any of the measured sites. BMD=bone mineral density. 3-month ROMO=3 months of romosozumab followed by 9 months of denosumab. 12-month ROMO=12 months of romosozumab.

The changes in BMD in the two treatment groups are shown in figure 2 and table 2. The mean 12-month change in BMD at the total hip was 5·7% (SD 3·3) in the 3-month ROMO group and 6·0% [3·2] in the 12-month ROMO group. This between-group difference in total hip BMD met the prespecified non-inferiority threshold as the 90% CI for the between-group difference was −1·2 to 1·9. The p value for the between-group difference in total hip BMD was 0·644.

The mean change in femoral neck BMD was 5·0% (SD 6·2) in the 3-month ROMO group and 6·3% (4·8) in the 12-month ROMO group (p=0·29 for the between-group comparison), whereas the mean change in lumbar spine BMD was 10·6% (5·4) in the 3-month ROMO group and 12·5 (4·4) in the 12-month ROMO group (p=0·184 for between-group comparison). The mean change in distal radius BMD was −0·3% (2·6) in the 3-month ROMO group and −1·5% (3·0) in the 12-month ROMO group (p=0·079 for between-group comparison).

**Figure 3:**
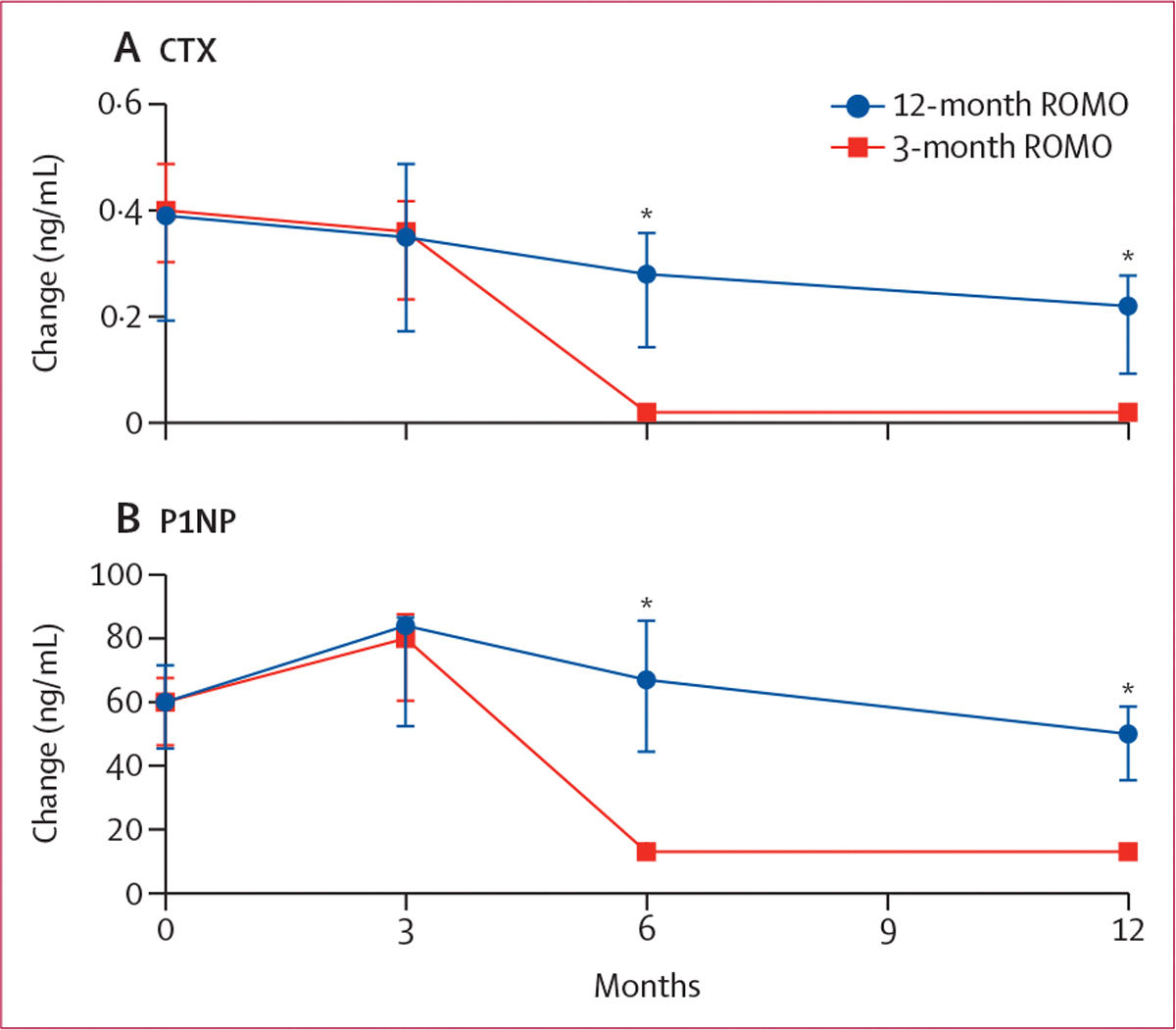
Change in serum markers of bone remodeling over the 12-month treatment period Mean absolute change from baseline in β-isomer of CTX (A) and P1NP (B) in the two treatment groups. Error bars indicate the 95% CIs (some error bars are contained within the symbol). Asterisks indicate a statistically significant between-group difference at that timepoint. 3-month ROMO=3 months of romosozumab followed by 9 months of denosumab. 12-month ROMO=12 months of romosozumab. CTX=C-terminal telopeptide of type I collagen. P1NP=procollagen type I N-terminal propeptide.

The changes in CTX and P1NP are shown in figure 3. P1NP is a measure of osteoblastic bone formation and CTX is a measure of osteoclastic bone resorption. P1NP increased in the cohort as a whole between month 0 and month 3 (p=0·0001) but no between-group differences were observed during the initial 3 months in either marker (when all participants were receiving the same intervention). Both markers differed between groups at all other timepoints (p<0·0001 for all comparisons except for p=0·0009 for CTX at month 6) as the group receiving denosumab exhibited greater suppression of both bone resorption and bone formation.

Adverse events are shown in table 3. Serious adverse events occurred in three participants, all in the 12-month ROMO group, and were deemed unrelated to the study treatment. These events were one participant being diagnosed with renal carcinoma, pneumonia, and deep venous thrombosis, one participant diagnosed with breast cancer, and one participant admitted to hospital for a small bowel obstruction. Four participants sustained non-vertebral fractures during the study (three in the 3-month ROMO group and one in the 12-month ROMO group). No participant died, had myocardial infarction, stroke, unstable angina, osteonecrosis of the jaw, or atypical femoral fracture.

## Discussion

In this study of postmenopausal women at high risk of fracture, we found that 3 months of romosozumab followed by 9 months of denosumab was non-inferior to 12 months of romosozumab in increasing total hip BMD. BMD changes at other sites were also similar between treatment groups. To our knowledge, this is the first randomised controlled clinical trial assessing the efficacy of a shorter romosozumab course in any population. In a retrospective, observational analysis of 169 women published as an abstract, BMD gains with 6 months of romosozumab followed by 6 months of denosumab were similar to gains observed with 12 months of romosozumab.^16^ Similar results were also reported in a retrospective 26-patient case series.^17^

The increases in BMD and changes in bone turnover observed in the 12-month ROMO group in this study are consistent with previous studies of romosozumab in postmenopausal women. Bone formation markers increase within days of romosozumab initiation but return towards baseline within weeks, whereas suppression of bone resorption persists throughout the 12-month course.^6,8,9^

Notably, the most substantial gains in BMD among patients on romosozumab occur during the first 6 months of treatment, with more modest increases thereafter. For example, in the original phase 2 study of romosozumab, total hip BMD increased by 2·9% in the first 6 months and only 1·1% in months 6–12.^9^ This pattern might at least partly reflect an early rapid modelling-based bone formation phase, followed by mineralisation of this newly formed bone during sustained antiresorptive activity. Similar patterns were observed in our previous DATA-Switch trial,^18^ in which transitioning from the osteoanabolic drug teriparatide to denosumab was associated with accelerated gains in BMD.

The clinical consequences of these findings are potentially substantial. Using romosozumab for 12 months is costly, requires monthly visits to a medical facility, and might be associated with an increase in cardiovascular risk. Allowing for an equally efficacious shorter course, aimed specifically to take advantage of the unique early osteoanabolic effects of romosozumab, could allow for more patients being treated because of reduced inconvenience and lower cost. Notably, a case-control study reported that incomplete insurance coverage was a major contributor to romosozumab non-adherence, the rate of which exceeds 50%.^13^ Moreover, patients might be more comfortable with taking romosozumab for a shorter period of time because of the potential of reduced cardiovascular risk as has been reported in some but not all previous studies of 12 months of romosozumab.^6,7,11^ In this regard, it should be noted that in the registration trials of romosozumab (all of which included transitioning to either alendronate or denosumab), the between-group incidence of major cardiovascular adverse events was similar after transition, suggesting that cardiovascular risk associated with romosozumab, if it exists, would wane quickly after discontinuation.^6,7^

The efficacy of denosumab when used after osteoanabolic therapy is not unique to the current study design.^19^ Indeed, current standard of care is that antiresorptive therapy be used after all osteoanabolic therapies to preserve gains in bone mass.^20^ Notably, in the phase 3 studies of romosozumab, both denosumab and alendronate have been shown to further increase BMD and maintain fracture risk reduction.^6,7^ Whether the long-term effects of other antiresorptive agents, including bisphosphonates, would be similar to denosumab when used after 3 months of romosozumab, remains to be understood given that denosumab’s suppression of bone remodelling is greater than that of other antiresorptive agents.^21,22^ Additionally, unlike bisphosphonates, denosumab might also preserve modelling-based bone formation in cortical bone.^23,24^

A limitation of this study is that its modest size did not allow for the assessment of anti-fracture efficacy directly. However, growing evidence supports the use of changes in BMD, particularly at the total hip, as a surrogate for treatment-related fracture risk reduction.^15,25–27^ Perhaps most relevant to the current study, Vilaca and colleagues^28^ examined whether changes in total hip BMD after 12 months of treatment were associated with fracture risk reduction using individual patient data from 22 randomised, placebo-controlled, double-blind trials of osteoporosis medications and found a strong predictive relationship, concluding that BMD measurement intervals of 12 months could adequately assess the anti-fracture efficacy of therapies over all fracture types and serve as a valid efficacy endpoint. Another potential limitation of this study is the lack of a 12-month denosumab comparator. That said, the 12-month spine, total hip, and femoral neck BMD gains reported with denosumab in similar populations have consistently been more modest than those observed in both treatment groups in the present trial.^29,30^

In summary, 3 months of romosozumab followed by 9 months of denosumab was non-inferior to 12 months of romosozumab in increasing total hip BMD. The ability of both regimens to increase BMD at the femoral neck, lumbar spine, and distal radius was also similar. These results suggest that shorter romosozumab regimens followed by denosumab might offer similar overall efficacy while addressing practical limitations of the standard approach. This strategy could shift the cost–benefit landscape of romosozumab use, potentially improving access by reducing insurance restrictions, increasing patient adherence, and enhancing safety perceptions. Together, these advantages might expand the reach of this uniquely effective osteoporosis therapy to a broader population of patients in need.

## Supplementary Material

MMC1

## Figures and Tables

**Table 1:** Baseline characteristics

	3-month ROMO (n=24)	12-month ROMO (n=26)

Age, years	69·5 (3·8)	69·7 (5·1)
Height, cm	162·5 (4·5)	160·7 (7·1)
BMI, kg/m^2^	22·9 (4·4)	24·1 (3·6)
Female sex	24 (100%)	26 (100%)
Race		
Asian	1 (4%)	0 (0%)
Black or African American	0	2 (8%)
White	23 (96%)	24 (92%)
Ethnicity		
Hispanic or Latino	1 (4%)	1 (4%)
Not Hispanic or Latino	23 (96%)	25 (96%)
History of fragility fracture	12 (50%)	11 (42%)
Previous bisphosphonate use	6 (25%)	8 (31%)
25-hydroxyvitamin D concentration in serum, ng/mL	42·7 (15·1)	40·6 (17·6)
P1NP, ng/mL	60·1 (21·0)	60·1 (24·9)
B-CTX, ng/mL	0·40 (0·15)	0·38 (0·26)
Posterior-anterior lumbar spine BMD, g/cm^2^	0·842 (0·128)	0·806 (0·100)
Femoral neck BMD, g/cm^2^	0·606 (0·074)	0·619 (0·078)
Total hip BMD, g/cm2	0·718 (0·079)	0·744 (0·080)
Distal one-third radius BMD, g/cm^2^	0·575 (0·069)	0·567 (0·063)
Posterior-anterior lumbar spine dual x-ray T score	−1·8 (1·2)	−2·2 (0·9)
Femoral neck dual x-ray T score	−2·2 (0·7)	−2·1 (0·7)
Total hip dual x-ray T score	−1·8 (0·6)	−1·6 (0·6)
Distal one-third radius DXA T score	−2·0 (1·2)	−2·1 (1·1)
FRAX 10-year probability of hip fracture	2·9% (2·1–6·1)	3·8% (2·1–5·0)
FRAX 10-year probability of major osteoporotic fracture	14·5% (11·0–21·0)	15·5% (14·0–20·0)

Data are mean (SD) n (%), or median (95% CI). 3-month ROMO=3 months of romosozumab followed by 9 months of denosumab. 12-month ROMO=12 months of romosozumab. 25(OH)D=25-hydroxyvitamin D. B-CTX=β-isomer of C-terminal telopeptide of type I collagen. BMD=bone mineral density. DXA=dual x-ray absorptiometry. FRAX=Fracture Risk Assessment Tool. P1NP=procollagen type I N-terminal propeptide.

**Table 2:** 12-month mean percentage change in BMD by treatment

	12-month ROMO	3-month ROMO	12-month ROMO *vs* 3-month ROMO	CI for the difference*

Primary				
Total hip	6·0% (3·2)	5·7% (3·3)	0·4% (3·3)	−1·2 to 1·9
Secondary				
Femoral neck	6·3% (4·8)	5·0% (6·2)	1·3% (5·6)	−1·4 to 4·0
Lumbar spine	12·5% (4·4)	10·6% (5·4)	1·8% (5·0)	−1·1 to 4·8
Exploratory	−1·5% (3·0)	−0·3% (2·6)	−1·2% (2·8)	−2·5 to 0·2
Distal radius				

Data are mean (SD), unless otherwise indicated. 3-month ROMO=3 months of romosozumab followed by 9 months of denosumab. 12-month ROMO=12 months of romosozumab. BMD=bone mineral density.

* 90% CIs for non-inferiority inference for total hip BMD (primary endpoint) and 95% CIs for the secondary endpoints, which were tested for between-group differences.

**Table 3:** Adverse events during treatment

	3-month ROMO (n=24)	12-month ROMO (n=26)

Any adverse event during treatment	21 (88%)	21 (81%)
Deaths	0	0
Any serious adverse event during treatment	0	3 (12%)
Fracture during treatment	3 (13%)	1 (4%)
Discontinuations due to adverse events	0	2 (8%)
Adverse events occurring in >10% of patients		
Back pain	6 (25%)	4 (15%)
Cough	7 (29%)	2 (8%)
Fatigue	3 (13%)	4 (15%)
Headache	5 (21%)	3 (12%)
Joint pain	3 (13%)	7 (27%)
Muscle cramps	5 (21%)	4 (15%)
Muscle pain	5 (21%)	5 (19%)
Palpitations	3 (13%)	3 (12%)
Paresthesia	3 (13%)	1 (4%)
Reaction at injection site	1 (4%)	5 (19%)
Rhinorrhea	3 (13%)	2 (8%)
Skin rash	5 (21%)	0
Swelling	3 (13%)	0

Data are n (%). 3-month ROMO=3 months of romosozumab followed by 9 months of denosumab. 12-month ROMO=12 months of romosozumab.

## Data Availability

The data that support the findings of this study are available from the corresponding author upon reasonable request.

## Research in context

### Evidence before this study

Before this randomised controlled trial was initiated, we searched PubMed for studies on romosozumab treatment in postmenopausal osteoporosis including search terms “postmenopausal osteoporosis”, “osteoanabolic”, and “romosozumab”. We included studies published from database inception until Feb 1, 2021). We found no previous randomised clinical trials that investigated the efficacy of shorter courses of romosozumab.

### Added value of this study

As the first randomised controlled trial to investigate the comparative efficacy of a short course of romosozumab (followed by denosumab), this non-inferiority study showed that in postmenopausal women at high risk of fracture, 3 months of romosozumab followed by 9 months of denosumab was non-inferior to 12 months of romosozumab in increasing total hip bone mineral density (BMD). The study also reports that the BMD changes at other anatomic sites (such as the femoral neck, wrist, and spine) were similar. This trial provides support for the use of shorter courses of romosozumab in this population.

### Implications of all the available evidence

Romosozumab is a unique osteoporosis therapy with both anabolic and antiresorptive effects and represents an important treatment option for postmenopausal osteoporosis, particularly in patients with established or severe disease. However, the drug’s use is limited by high cost, payer restrictions, the need for monthly clinic-administered injections, and a possible association with increased cardiovascular risk. The results of this randomised controlled trial provide rationale for a shorter, 3-month course of romosozumab compared with the standard 12-month regimen, thereby reducing cost and injection burden while potentially limiting cardiovascular risk.
