# Supplementary material for: 3 months vs 12 months of romosozumab for postmenopausal osteoporosis (LIDA): an open-label, non-inferiority, randomised controlled trial
Source: Lancet Diabetes Endocrinol. Author manuscript; Available in PMC 2026 Mar 17. (PMC12993474; doi:10.1016/S2213-8587(25)00319-5)
Supplement: MMC1 [file NIHMS2145497-supplement-MMC1.pdf]

THE LANCET
Diabetes & Endocrinology

Supplementary appendix

This appendix formed part of the original submission and has been peer reviewed. We post it as supplied by the authors.

Supplement to: Leder BZ, Ramchand SK, Jordan M, et al. 3 months *vs* 12 months of romosozumab for postmenopausal osteoporosis (LIDA): an open-label, non-inferiority, randomised controlled trial. *Lancet Diabetes Endocrinol* 2026; published online Jan 29. https://doi.org/10.1016/S2213-8587(25)00319-5.

Mass General Brigham

Institutional Review Board
Intervention/Interaction Detailed Protocol

Principal Investigator: Benjamin Leder, MD

Project Title: Limited-duration anabolic therapy in postmenopausal osteoporosis

Version Date: August 22, 2022

1. Background and Significance

(A) Clinical Significance and Biological Relevance

Osteoporosis affects over 20 million Americans leading to 1.5 million fragility fractures and 300,000 hip fractures every year (1, 2). The most commonly used antiresorptive and anabolic medications can at best increase bone mineral density (BMD) modestly and decrease non-spine fracture rates by 20-40% (3-7):

	2-yr increase spine BMD	2-yr increase total hip BMD	RRR of spine fx	RRR of non-spine fx
Alendronate	5%	3%	53%	20%
Zoledronic acid	5-6%	3-4%	70%	25%
Denosumab	6-8%	3-4%	68%	20%
Teriparatide	10%	2-3%	65-80%	30-48%
Abaloparatide	10%	3-3.5%	86%	43%

- RRR = relative risk reduction
- Antiresorptive therapies in black
- Anabolic therapies in blue

Currently, most guidelines suggest that antiresorptive osteoporosis medications should be used for only 3-5 consecutive years in most patients (8, 9). Anabolic agents are used for 12-24 months per FDA guidelines. Thus, patients with established osteoporosis are often treated with several medications in sequence. And while most patients with osteoporosis in the U.S. are currently initially treated with antiresorptive drugs, it has become increasingly well-understood that the initial use of an anabolic agent followed by an antiresorptive medication increases BMD more than using these drugs in the opposite sequence (10-15).

Romosozumab, the most recently approved osteoporosis medication, increases bone mass through both osteoanabolic and antiresorptive effects (16). Clinically, romosozumab increases trabecular and cortical BMD more rapidly than any currently available single agent and reduces vertebral fractures compared to placebo and both vertebral and non-vertebral fractures when compared to the most commonly-used osteoporosis medication, alendronate (17, 18). Despite its unequivocal efficacy, however, the use of romosozumab is currently limited by its inconvenient dosing (2 SC injections monthly for 12 months), high cost ($21,900 for a 12-month course of romosozumab, roughly 10X higher than denosumab), and cardiovascular safety concerns that have necessitated a "black-box" warning. Thus, developing romosozumab regimens that can expand the spectrum of appropriate candidates for the drug without compromising efficacy would be transformative.

Romosozumab mechanism of action: Sclerostin is an osteocyte-secreted protein that acts as an extracellular inhibitor of canonical Wnt signaling by binding to lipoprotein receptor-related proteins-4, 5 and 6 (19). The effects of sclerostin on bone were first identified through studies of

patients with inactivating mutations in the gene encoding sclerostin, who presented with bone overgrowth (20-25). Sclerostin's dominant function is to inhibit the proliferation, differentiation, and survival of osteoblasts (hence inhibiting bone formation) but has also been shown to up-regulate osteocytic RANKL synthesis, thereby stimulating bone resorption (26, 27). Romosozumab is a monoclonal antibody that inhibits osteocyte-derived sclerostin and its observed anabolic and antiresorptive effects fit well with the known effects of sclerostin in human physiology. Romosozumab's stimulation of new bone formation, however, is transient and not sustained beyond 3 months despite continued administration (17, 28). Indeed, when bone biopsies are performed in postmenopausal women after 2-months of drug exposure, bone formation rates are increased and bone resorption suppressed whereas biopsies taken after 12-months of romosozumab demonstrate suppression of both formation and resorption (16). Finally, in a 3-month placebo controlled trial of 48 men and women, romosozumab rapidly and significantly improved trabecular volumetric BMD and separation as well as cortical thickness and estimated bone strength (assessed by high-resolution QCT of the spine)(29).

Romosozumab clinical efficacy when used in sequence with denosumab: Denosumab is a fully human monoclonal antibody that specifically binds to receptor activator of nuclear factor-κB ligand (RANKL)(30). It inhibits bone resorption by interfering with the binding of RANKL to its receptor on pre-osteoclasts and osteoclasts, the final common pathway leading to osteoclast activation and bone resorption (31, 32). Denosumab suppresses bone turnover more than intravenous and oral BPs and more than romosozumab (33-35). When romosozumab is discontinued, BMD promptly reverts towards baseline unless immediately followed by either a bisphosphonate or denosumab (18, 36). The sequential use of romosozumab followed by denosumab appears to be particularly efficacious. For example, when 12-months of romosozumab is followed by 12 months of denosumab, spine and hip BMD increase by 17.6% and 8.8%, respectively, and spine fractures were reduced by 75% compared to 12-months of placebo followed by 12-months of denosumab (17).

Romosozumab Safety Considerations: The data underlying the romosozumab's safety issues are discussed in detail in the *Prior Studies and Rationale* section below. Briefly, the most worrisome safety issue stems from the higher rate of major adverse cardiovascular events or MACE (a composite endpoint of cardiovascular death, nonfatal myocardial infarction and nonfatal stroke) in postmenopausal women treated with romosozumab compared to those treated with alendronate as well as in a smaller placebo-controlled trial in men (17, 37).

2. Specific Aims and Objectives

The aim of this clinical trial is to test the following hypothesis: In postmenopausal women with osteoporosis, 3-months of romosozumab followed by 9 months of the RANK ligand inhibitor, denosumab, will result in non-inferior increases in BMD, improvements in skeletal microstructure, and increases in estimated bone strength compared to 12-months of romosozumab.

3. General Description of Study Design

The study will be a randomized, open-label, proof-of-principal clinical trial (investigators assessing all endpoints will be blinded to group assignment). **46 female volunteers** will be recruited for the study and randomized to 1 of 2 groups as shown in the figure below:

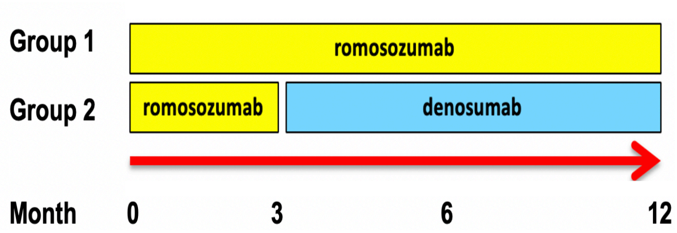

4. Subject Selection

Inclusion Criteria: The inclusion criteria were developed to conform with the FDA-approved indications for romosozumab and denosumab. The lack of a placebo group allows for recruiting this high-risk population.

1) Women age> 45

2) Postmenopausal by either of the following criteria:
   > 36 months since last spontaneous menses.
   > 36 months since hysterectomy, plus serum FSH > 40 units / liter if < 60 years.

3) High risk of fracture defined as one of the following:
a. Prior history of osteoporotic fracture
b. Hip or spine BMD T-score ≤ -2.5

Exclusion Criteria

Prior therapy exclusions

1. current use or use in the past 12 months of oral bisphosphonates or denosumab
2. current use or use within the past 3 months of estrogens, SERMs, or calcitonin.
3. use of oral or parenteral glucocorticoids for more than 14 days within the past 6 months
4. any current or previous use of romosozumab or strontium
5. current use or use within the past 3 years of teriparatide, abaloparatide, or any parenteral bisphosphonate.

Other exclusions

1. Confirmed serum alkaline greater than 2 times the upper normal limit.
2. Stage 4 or 5 chronic kidney disease (GFR less than 30)
3. Hypercalcemia (Ca greater than 10.5 mg/dL)
4. Hypocalcemia (Ca less than 8.8 mg/dL)
5. Elevated blood PTH (intact PTH greater than 77 pg/mL)
6. Serum 25-OH vitamin D less than 20 ng/mL*
7. HCT less than 32%.
8. History of malignancy (except basal cell carcinoma).
9. Significant pulmonary disease
10. History of myocardial infarction or stroke within the preceding year.
11. History of unstable angina or transient ischemic attack in the past year.
12. Current atrial fibrillation.
13. Any health condition that, in the opinion of the study physician, significantly increases the risk of cardiovascular events.
14. Major psychiatric disease that in the opinion of the investigator would preclude the subject

from providing adequate informed consent or completing the protocol procedures.

15. Excessive alcohol use or substance abuse that in the opinion of the investigator would preclude the subject from providing adequate informed consent or completing the protocol procedures.
16. Uncontrolled eczema
17. Congenital or acquired bone disease other than osteoporosis.
18. Known sensitivity to denosumab or any of its excipients.
19. Known sensitivity to romosozumab or any of its excipients.
20. History of osteonecrosis of the jaw, extensive dental work involving extraction or dental implants in the past 3 months, or extensive dental work planned in the upcoming 12 months.

*If a subject, on initial screening, has a 25-OH vitamin D concentration less than 20 ng/mL, a period of vitamin D replacement and re-screening of 25-OH vitamin D will be permitted. For subjects not taking vitamin D supplementation, vitamin D will be replaced according to the table below. For subjects already taking vitamin D supplementation, replacement will be based on the advice of the study physician, taking into consideration the subject's compliance with their current vitamin D supplementation, the duration of supplementation, and any issues with oral absorption. Re-screening of 25-OH vitamin D will be performed after a minimum of 3 weeks of replacement.

Serum 25-OH vitamin D (ng/mL)	Vitamin D3 Supplementation Dose (IU) and Frequency
12-20	3,000 daily
<12	5,000 daily

<u>Recruitment:</u>
Over the past decade, we have repeatedly demonstrated the ability to recruit postmenopausal women with osteoporosis in a timely fashion and have consistently met enrollment goals. In the DATA studies, we recruited larger cohorts of women with similar entry criteria extremely efficiently. In our currently-enrolling R01-funded study entitled Mechanisms Underlying the Bone Modeling Effects of Combined Anabolic/Antiresorptive Administration, in which 36 postmenopausal osteoporotic women are being enrolled for a study that involves bone biopsy, we have met our recruitment goals despite the current COVID crises. In the present study, volunteers will be recruited through methods that we have used successfully in the past. These methods include recruitment from endocrine and internal medicine clinics, email announcements, and mailings to targeted populations.

Recruitment flyers will be posted in approved locations throughout the MGH (including the MGH Bone Mineral Density Center) and email announcements will be sent and posted via Rally with Mass General Brigham.

Subjects may be recruited from the clinical practice (MGH Endocrine Associates) of the principal investigator and co-investigator(s). To avoid potential coercion, after the investigator has briefly presented the study, the participant will be asked if they may be re-contacted for a more in-depth phone call from another study physician. Additionally, informed consent for screening will be completed by a research coordinator/study physician and informed consent for the main study will be completed by a study physician who is not a provider for each of the patients/subjects of this recruitment method.

We aim to enroll a diverse subject population. We will ensure proper minority participation by using the recruitment strategies that focus on areas within the city of Boston and surrounding towns with significant ethnic diversity. These strategies have been successful at achieving significant African-American and Latino participation in previous studies by our group

Retention efforts will include regular communication between study staff (including study physicians) and our study volunteers with frequent reminder emails and calls and consistent same-day responses to subject calls or emails. Additionally, we have yearly social events for all participants that have been extremely well-received. These retention methods have proven to be successful in the past. For example, the cumulative 1-year retention rate in the DATA and DATA-HD studies was >90%.

5. Subject Enrollment

Members of research staff will screen all interested subjects over the telephone. If subjects meet initial criteria and are interested in the study, a member of the study staff will schedule a screening visit wherein the subject will sign a separate "screening only" consent form. The signing of the screening consent will take place in the exam room at the Bone Density Center at MGH (10 Emerson Place). The screen consent may be obtained by a study physician or study coordinator. We plan to enroll about 250 subjects in the screening part of this study at MGH in order to enroll about 46 subjects in the main part of the study at MGH.

This consent form will allow for a blood draw and bone mineral density testing. Prior to signing this screening only consent form, subjects will be offered the option to speak with a physician investigator if they wish or if they have questions that can't be answered by the research staff. At this visit, bone mineral density of the spine and hip will be obtained along with the necessary recruitment labs. If the subject meets the above BMD and laboratory criteria, she will then be scheduled for visit 1.

The full consent form will be signed in the presence of a study physician on TH-10 at MGH or at the MGH Bone Density Center (10 Emerson Place). If subjects require more time to consider participating after reading the consent form, the subject will not be enrolled, and the study visit will not continue. If the subject is ready to sign the consent form, study procedures will then begin.

For those subjects who do not speak English, we will use the IRB template "Short form" for non-English speakers and arrange for a hospital translator to be present, either in person or by telephone, during all consent procedures. If using a telephonic interpreter, study staff will note the company name, the interpreter ID number, the date, and time.

6. STUDY PROCEDURES

Groups and randomization: Subjects are screened and, if eligible, will be invited to participate in the main study. Once a subject has signed the main study consent form, they are considered enrolled in the study and are then assigned to one of the 2 groups by computer-generated assignment using a randomly varying blocking scheme. Subjects will be stratified by age (above or below 65) and prior oral bisphosphonate use (yes or no).

Group assignment:

Group 1: romosozumab 210 mg SC every month for 12 months.
Group 2: romosozumab 210 mg SC every month for 3 months followed by denosumab 60 mg SC at month 3 and again at month 9.

Study procedures:

Screen Visit:
At this screen visit, bone mineral density of the spine and hip will be obtained, and the following will be measured in serum or blood:

- PTH, 25-OH vitamin D, CBC, and comprehensive chemistry panel (serum glucose, BUN, Cr, eGFR, Na, K, Cl, Ca, total protein, albumin, bilirubin, ALP, ALT, AST)
- FSH (if necessary due to prior hysterectomy and age less than 60 years)
- A historic DXA performed for clinical indications or undergone as part of another protocol may be used if the DXA scan is performed within 6 months of the Screen Visit.

Note: If a subject's screen eligibility labs contain an abnormal result/s that, in the opinion of the investigator, should be repeated, the subject will be invited to have the abnormal test/s repeated with study staff or study staff will recommend that the subject have the test/s repeated with the subject's clinical care team. If greater than 12 months have lapsed between the subject's screening visit and their first main study visit, study staff will invite subjects to undergo a re-screen for accuracy and safety purposes.
The study period lasts 12 months.

Visit 1:
If the subject meets the above BMD and laboratory criteria, she will then be scheduled for Visit #1. At this visit, a study physician will obtain written informed consent for participation in the complete study. If subjects require more time to consider participating after reading the main study ICF the subject will not be enrolled, and the study visit will not continue. If the subject has signed the main study ICF, the study physician will then perform a history and physical. If all inclusion/exclusion criteria are met, subjects will then proceed with the rest of visit 1 procedures and the subsequent visits as listed in the table below (Study Visit Schedule). If there is a delay in obtaining insurance approval for romosozumab, subjects may be asked to return shortly after visit 1 to receive their first dose of romosozumab.

Visit 2-4:
Injection visits will occur monthly for romosozumab and at months 3 and 9 for denosumab.

Subjects will be seen at MGH prior to 10 a.m. and will be instructed to be fasting. The following blood/serum tests will be measured based on the schema and visit schedule below:

- Comprehensive chemistry panel (serum glucose, BUN, Cr, eGFR, Na, K, Cl, Ca, total protein, albumin, bilirubin, ALP, ALT, AST), 25-OH vitamin D, PTH and CBC
- Serum CTX (pooled* assay at end of study)
- Serum PINP (pooled* assay at end of study)

*Samples for CTX and PINP will be run in a batch assay at the conclusion of the study.

If a subject's visit labs contain an abnormal result/s that, in the opinion of the investigator, should be repeated, the subject will be invited to have the abnormal test/s repeated with study

staff or study staff will recommend that the subject have the test/s repeated with the subject's clinical care team.

After which the subjects will be given breakfast, and then have the following procedures per table below:

Study Visit Schedule

**Month**	**0**	**1**	**2**	**3**	**4**	**5**	**6**	**7**	**8**	**9**	**10**	**11**	**12**
**Visit**	**1**			**2**			**3**						**4**
Inclusion/Exclusion Criteria	X												
ICF	X												
Medical History	X												
Medication History	X												
Vitals	X			X			X						X
romosozumab injection (per group assignment)	X	X	X	X	X	X	X	X	X	X	X	X	
denosumab Injection (per group assignment)				X						X			
Lab Work [Chemistries, 25OH vit D, PTH, and CBC]	X			X			X						X
Lab Work [Bone Turnover Markers*]	X			X			X						X
DXA (spine (including TBS), hip, and radius)	X			X			X						X
HR-pQCT (radius and tibia)	X												X

*Serum carboxy-terminal collagen crosslinks (CTX) and procollagen type I pro-peptides (PINP)
All subjects reporting dietary intake <1200mg of calcium/day will be given calcium supplementation (600mg) with Vit D (400IU). Subjects with a dietary intake >1200 mg will receive Vit D only (minimum dose of 400IU).

Investigators involved in the interpretation of imaging studies will not be aware of group assignment.

The study statistician (Dr. Hang Lee) will maintain the randomization assignment list and will coordinate group assignments. Subjects who elect to drop out of the study before the last visit will be asked to do an early termination visit. The markers of bone turnover will be performed on frozen serum at study conclusion (all samples for any individual subject will be performed in the same assay). Samples will be stored at -80C.

Study Drugs: Both studies drugs will be administered as per the FDA label and at the FDA-approved doses. Both romosozumab and denosumab will be obtained through the subject's medical insurance and will be obtained, stored, and dispensed as per standard MGB outpatient clinic protocol. Injections will be administered by a study physician or study staff with the appropriate training and education (e.g. a registered nurse).

Endpoints: **The primary endpoint of the proposed clinical trial is the change in *DXA-derived areal BMD of the total hip month 0-12.***

Secondary endpoints include:
• Areal BMD of the femoral neck
• Areal BMD of the lumbar spine

Exploratory endpoints include:
• Serum markers of bone turnover (CTX, P1NP)
• BMD of the distal radius
• Parameters of trabecular microarchitecture of the distal radius and tibia (HR-pQCT)
• Cortical thickness and cortical porosity of the distal radius and tibia (HR-pQCT)
• FEA-estimated bone strength of the radius and tibia (HR-pQCT)
• Trabecular bone score of the lumbar spine (DXA)

Incidental Findings: If a subject's visit labs contain an abnormal result/s that, in the opinion of the investigator, should be repeated, the subject will be invited to have the abnormal test/s repeated with study staff or study staff will recommend that the subject have the test/s repeated with the subject's clinical care team.

Subjects will be compensated for their time with $25 after each visit and $20 after each injection only visit for a total of $160 or $280 depending on treatment group. Subjects will not be compensated for the screen visit and will be mailed a copy of their screen visit bone density results.

Parking vouchers will be provided to subjects at each visit, including the screening visit and each time they come to MGH to receive either romosozumab or denosumab.

7. Risks and Discomforts

Medication Risks

Romosozumab: Romosozumab is an FDA approved medication used to treat postmenopausal women with osteoporosis who are at high risk of fracture. The most commonly reported side effects in the clinical trials of romosozumab (reported in >2% of treated women) include arthralgia, headache, muscle spasm, edema, asthenia, neck pain, insomnia, and paresthesia. *Major Adverse Cardiovascular Events (MACE):* There have been 2 large phase 3 clinical trials of romosozumab, one was placebo controlled and the other an active comparator study versus alendronate (17, 22). During the 12-month double-blind treatment period of the placebo-controlled study, no difference in MACE were observed between treated and placebo patients (myocardial infarction occurred in 9 women (0.3%) in the romosozumab group and 8 (0.2%) women in the placebo group; stroke occurred in 8 women (0.2%) in the romosozumab group and 10 (0.3%) women in the placebo group). In the 12-month double-blind treatment period of the active-controlled trial, however, myocardial infarction occurred in 16 women (0.8%) in the romosozumab group and 5 (0.2%) women in the alendronate group; stroke occurred in 13 women (0.6%) in the romosozumab group and 7 (0.3%) women in the alendronate group. The number of women with positively adjudicated MACE was 41 (2.0%) in the romosozumab group and 22 (1.1%) in the alendronate group (HR = 1.87, 95% confidence interval 1.11-3.14). To address this issue, the FDA's Black- Box warning reads: "romosozumab *may increase the risk*

of myocardial infarction, stroke, and cardiovascular death. It should not be initiated in patients who have had a myocardial infarction or stroke within the preceding year. Consider whether the benefits outweigh the risks in patients with other
therapy, ROMO should be discontinued". Our exclusion criteria conform with these label recommendations. Atypical femur fractures and osteonecrosis of the jaw have both been observed rarely with romosozumab though causality has not been established. Subjects will be informed that use of romosozumab in this study is off-label due to the abbreviated duration of use.

Denosumab: Denosumab is an FDA approved medication used to treat osteoporosis in various populations, including postmenopausal women. More than 13,500 patients have been treated with denosumab in clinical studies, and it is generally well tolerated. In clinical studies, it has been reported that denosumab may uncommonly produce the following side-effects: eczema, serious skin infections, low blood calcium (which can cause tingling in the fingers or around the mouth, muscle cramps, or abnormal heart rate), pain in the joints or extremities, high blood cholesterol, dizziness, cough, difficulty emptying the bladder, and decreased skin sensation. Hypocalcemia has been very rarely reported in the absence of renal failure. In a clinical trial of over 7800 women with postmenopausal osteoporosis, serious infections leading to hospitalization were reported more frequently with denosumab than placebo (serious skin infections, as well as infections of the abdomen, urinary tract, ear, as well as endocarditis were more frequent with denosumab than placebo). Of note, the incidence of opportunistic infections was balanced between placebo and treated groups and the overall incidence of infections was similar between the treatment groups. Osteonecrosis of the jaw and atypical femur fractures have been reported in patients treated with denosumab but its incidence, while rare, remains unclear. The short-term (12 month) design of this study reduces the risk of these side effects. Subjects will be informed that use of denosumab in this study is off-label as the inclusion criteria for being at high risk of fracture does not necessarily require a diagnosis of osteoporosis as defined by bone mineral density criteria alone.

Discontinuing Denosumab: Following the discontinuation of denosumab, markers of bone resorption increase above pretreatment values, BMD decreases, and the risk of vertebral fracture increases, including the risk of multiple vertebral fractures. These vertebral fractures occurred as early as 7 months (on average 19 months) after the last dose of denosumab. For this reason, it is now recommended that all patients discontinuing denosumab transition to an alternative antiresorptive therapy. In our prior studies with denosumab, including the DATA studies, we have successfully used multiple methods to ensure that upon study completion antiresorptive therapy is not stopped abruptly but rather is either continued by their treating physician or through specialist referral. Specifically, at the initial consent as well as subsequent study visits, regardless of group assignment, subjects will be counseled regarding the increased risk of vertebral fracture when denosumab is discontinued. They will also be counseled that BMD gains with romosozumab are rapidly reversible in the absence of a transition to antiresorptive therapy. At the final study visit, all study participants will meet with a study physician who will review the experience of the participant during the study and explain to the participant that it is imperative that they arrange follow up with their primary care physician within 1-2 months of finishing the study so that a treatment plan can be initiated. If the subject does not have a primary care physician, subjects will be encouraged to see a physician with some expertise in the treatment of osteoporosis and offered the opportunity to schedule an appointment with such a physician at Massachusetts General Hospital. With the subject's permission, a letter will be sent to their treating physician which will including information regarding the risks of abruptly discontinuing denosumab as well as the results of all relevant

investigations from the study. Study participants will be reminded of this information via a follow-up phone call 2 weeks after exit from the study to ensure that they have received appropriate follow-up.

Radiation Risks:
Over the 12-month study period, subjects will receive the following imaging studies:

Study Type	Total Number	Radiation exposure per scan
DXA of the lumbar spine, hip, and radius.	5 (including screen)	1 mrem
HR-pQCT of the distal tibia and radius	2	<0.5 mrem

The overall total dose for the entire 12-month study is therefore <10 mrem. This amount of radiation is equal to the background radiation one is exposed to from the earth and sky in one month.

Privacy Risks:

All subjects will be informed of their rights under the Health Insurance and Portability and Accountability Act of 1996 per federal law and hospital policy. The consent form contains an IRB-approved template that describes what protected health information from the research study may be used or shared with others. Data collected strictly for research purposes are stored in locked files and on computers with passwords required for access.

8. Benefits

The proposed study is evaluating a sequential treatment approach that has the potential to significantly improve upon current osteoporosis treatment. The 2 drugs administered as part of this proposal are FDA- approved and are being used per the labelled indications and at the approved doses. Regardless of group assignment, patients will benefit from the osteoporosis treatment that they receive during the course of this study. Subjects may also benefit from the screening tests performed, whether or not they ultimately enroll in the trial.

9. Statistical Analysis

Statistical Analysis: All analyses will follow ARRIVE guidelines (e.g., randomize and blind, report attrition) and NIH guidelines on rigor (NOT-OD016-011). Analyses will use all study participants who received the study medications and completed at least 1 post-baseline visit (modified intention to treat analysis). Baseline demographic- and clinical characteristics will be summarized using descriptive statistics and between-group differences will be tested using independent samples t-test for continuous measurements and Chi-square test for categorical outcomes. We will conduct a non-inferiority test for each primary and secondary endpoint by examining if the 80% confidence interval of the difference in the expected 12-month changes that is estimated from the random-intercept longitudinal linear effects model resides within ± M, where M is the prespecified non- inferiority margin described below.

Setting the non-inferiority margin (M): The determination of non-inferiority margin can be done in several ways. Traditionally, this margin is set based on a subjective determination of physiological relevance and clinical judgement. Other methods are based on mathematical concepts (see: Althunian et al *Br J Clin Pharmacol.* 2017;83(8):1636-42., Althunian et al. *Trials.* 2017;18(1):107). These include:

1) Fixed margin method: which uses a value ranging from 50 to 75% of the difference between the values found in control and placebo.

2) FDA advised method which uses 50% of the lower limit of the 95% confidence interval of the difference between the values found in the control and in the placebo groups.

Based on the phase 3 placebo-controlled trial of romosozumab (see reference 17), we calculated the following for the primary endpoint of total hip BMD.
• Using method #1 and the M value would be between 1.8 and 3.5% (effect size = 6.9%).
• Using method #2 and the M value would be 2.8% (lower CI = 5.6%).

We have set the non-inferiority margin (M) at a conservative value of 2%, requiring a sample size of 18 trial participants per Arm as shown below. Of note, this M value is not only consistent with the mathematical guidelines discussed above but is also lower than what would generally be considered clinically relevant. Additionally, the M value is significantly lower than both a mathematically and clinically-derived non-inferiority margin for lumbar spine BMD.

Safety Analysis: Adverse events will be recorded in RedCap from source documents and will be reviewed quarterly by the study team and PI. Our study is not sufficiently powered for any formal safety analysis.

Sample Size, Effect Size and Power: We calculated the necessary sample size based on the M value for our primary endpoint (total hip BMD), assuming similar standard deviations (SD) as those reported for change in BMD in the randomized placebo-controlled trial of romosozumab (3). The necessary sample size to attain 90% power at 5% Type-1 error rate of the non-inferiority test is shown below:

**Variable**	**Sample size per group**	**SD**	**M-value**
DXA total hip (% change)	**21**	**2.3**	**2**

We anticipate that there will be up to 10% of the study participants who cannot complete the 12 months visit. In order to accrue the required sample size to retain 90% power for the primary analysis at a 5% Type-1 error rate, we will enroll a total of 46 patients (23/Arm).

Missing Data: As described above, the modified intention to treat analysis using longitudinal linear mixed effects model with Restricted Maximum Likelihood (REML) method will be conducted. This analysis, without incorporating a multiple-imputation, will provide a treatment effect estimate which is consistent with that of the complete cases data if the missing data occurs at random (MAR). As a parallel analysis, we will carefully examine and interpret this result if it is close to that of a multiple-imputation based analysis under MAR assumption. We will also conduct a ‘complete case’ analysis and compare the results of this analysis with those of the REML using all available data and the data sets augmented by the multiple-imputation.

10. Monitoring and Quality Assurance

The proposed study will be monitored by the PI as well as our formal Data Safety Monitoring Board (DSMB) and our Institutional Review Board (IRB).

Statement of Compliance: The study will be conducted in compliance with the protocol, International Council for Harmonization/Good Clinical Practice (ICH/GCP) requirements, and applicable state, local, and federal regulatory requirements. All personnel involved in the conduct of this study have completed Human Subjects Protection and ICH/GCP training. The protocol, informed consent form(s), recruitment materials, and all participant materials will be submitted to the IRB for review and approval. Approval of both the protocol and the consent form must be obtained before any participant is enrolled. Any amendment to the protocol will require review and approval by the IRB before the changes are implemented to the study. A determination will be made regarding whether new consent needs to be obtained from participants who provided consent using a previously approved consent form.

Protocol deviations will be brought to the attention of the PI immediately upon study staff becoming aware that a deviation has occurred. Deviations will be scored as to whether they are minor or major. Any deviation that impacts subject safety will be reported to NIAMS and the DSMB, via the NCR Executive Secretary, within 48 hours and all events will be reported in the 6-monthly DSM reports. All major deviations will be reported to the IRB within 5 business days.

Definitions and Classifications of Protocol Deviations: Protocol deviations are any deviations from the IRB-approved protocol that are not approved prospectively by the IRB. Protocol deviations will be classified as either major or minor.

Minor Protocol Deviation**:** Minor protocol deviations are deviations that do not have the potential to negatively impact subjects, their willingness to participate, or data integrity. Minor deviations include, but are not limited to, protocol deviations such as out of window visits, missing tests/labs, missing original/signed consent form (copy exists), missing PI signature on consent form(s), use of expired/outdated consent form that includes all relevant information, over-enrollment, or failure to submit continuing review prior to expiration of IRB approval.

Major Protocol Deviation: Major protocol deviations are deviations from the IRB-approved protocol that have the potential to negatively impact subject safety, the integrity of study data (ability to draw conclusions from the study data), or subject's willingness to participate in the study.

Safety Monitoring: The PI or other study physician will review all data collection on a weekly basis to ensure completeness, accuracy and protocol compliance. Routine safety laboratory testing will be reviewed within 48 hours of their reporting. Any abnormal laboratory testing will be reviewed in real-time. Any abnormal laboratory value/s in the opinion of the PI believed to be related or possibly related to the study intervention or study drug will be reported in the Adverse Event (AE) log. The AE log will be reported at each DSMB meeting. Review of the rate of subject accrual and compliance with inclusion/exclusion criteria will occur weekly during the recruitment phase and then monthly to ensure that enough participants are being enrolled and that they meet eligibility criteria and the targeted ethnic diversity goals outlined in the grant proposal. The PI will be responsible for identifying, managing and reporting all reportable events (serious adverse events, adverse events and unanticipated problems), to the MGB IRB, the Food and Drug Administration (FDA), and the DSMB. All participating facilities (MGH core labs, MGH research pharmacy) are regularly monitored as part of their participation in the Harvard Clinical and Translational Science Center (CTSC).

Data Safety Monitoring Board: In addition to the safety procedures described above, a DSMB will be appointed by NIAMS. The DSMB members are chosen by NIAMS. The DSMB charter

will be forwarded when finalized. The DSMB will be responsible for ensuring scientific integrity and protecting the safety of study subjects. The DSMB will meet with the PI and relevant study staff every 6 months to review the study data. The DSMB will have access to all data and the authority to perform more formal interim analyses should there be a suggestion of increased adverse events occurring in either treated group. The DSMB will have the authority to recommend stopping the study if any safety concerns should arise. The DSMB will also review investigator and study staff performance (recruitment, retention, data forms, protocol adherence, and data quality).

Adverse Event Definitions:

Adverse Event (AE): An adverse event is any unfavorable and unintended diagnosis, sign (including an abnormal laboratory finding), symptom, or disease temporarily associated with the study intervention, which may or may not be related to the intervention. AEs include any new events not present during the pre-intervention period or events that were present during the pre-intervention period which increased in severity.

Serious Adverse Event (SAE): A serious adverse event is any untoward occurrence that results in death, is life-threatening, requires or prolongs hospitalization, causes persistent or significant disability/incapacity, results in congenital anomalies/birth defects, or, in the opinion of the investigators, represents other significant hazards or potentially serious harm to research participants or others.

Classification of an Adverse Event: All AEs and SAEs will be assessed by the study physician and assigned to the following categories:

Mild: Events require minimal or no treatment and do not interfere with the participant’s daily activities.

Moderate: Events result in a low level of inconvenience or concern with the therapeutic measures. Moderate events may cause some interference with functioning.

Severe: Events interrupt a participant’s usual daily activity and may require systemic drug therapy or other treatment. Severe events are usually potentially life-threatening or incapacitating. Of note, the term “severe” does not necessarily equate to “serious”.

Relationship to Study Intervention: All AEs will have their relationship to study intervention assessed by the clinician who examines and evaluates the participant based on the temporal relationship and his/her clinical judgment. The degree of certainty about causality will be graded using the categories below:

Definite: There is clear evidence to suggest a causal relationship, and other possible contributing factors can be ruled out. The clinical event, including an abnormal laboratory test result, occurs in a plausible time relationship to study intervention administration and cannot be explained by concurrent disease or other drugs or chemicals. The response to withdrawal of the study intervention (dechallenge) should be clinically plausible. The event must be pharmacologically or phenomenologically definitive, with use of a satisfactory rechallenge procedure if necessary.

Probable/Possible: There is evidence to suggest a causal relationship (e.g. the event occurred

within a reasonable time after administration of the trial medication), but other factors are present which may have contributed to the event (e.g. the participant's clinical condition, other concomitant events). However, there is greater than 50% certainty that the event is related to study intervention. The event follows a clinically reasonable response on withdrawal (dechallenge). Rechallenge information is not required to fulfill this definition. Although an AE may rate as "probable/possible" upon discovery, it can be flagged as requiring more information and later be upgraded to "definite," as appropriate.

Unrelated/Unlikely related: The AE is completely independent of study intervention administration, and/or evidence exists that the event is definitely or likely related to another etiology.

Expectedness: The study investigator will be responsible for determining whether an AE is expected or unexpected. An AE will be considered unexpected if the nature, severity, or frequency of the event is not consistent with the risk information previously described for the study intervention.

Definition of an Unanticipated Problem Involving Risks to Subjects or Others (UPIRTSO):
Any incident, experience or outcome that meets **all 3** of the following criteria:
1. Unexpected* in terms of nature, severity or frequency given (a) the research procedures that are described in the protocol-related documents and informed consent documents and (b) the characteristics of the subject population being studied.
2. Related or possibly related** to participation in the research
3. Suggests that the research places subjects or others at a greater risk of harm*** (including physical, psychological, economic, or social harm) than was previously known or recognized.

*Unexpected: the incident, experience, or outcome in terms of nature, severity, or frequency is not consistent with either:
(i) the known or foreseeable risk of events associated with the procedures involved in the research that are described in the (a) protocol related documents, any applicable investigator brochure, or the current IRB approved informed consent documents and (b) other relevant sources of information, such as product labeling and package inserts; or
(ii) the expected natural progression of any underlying disease, disorder, or condition of the subject(s) experiencing an adverse event and the subject's predisposing risk factor profile for the adverse event.

** Related or possibly related: Events that are determined to be at least partially caused by the procedures involved in the research. Events that are caused solely by an underlying disease, disorder or condition or events caused solely by other circumstances unrelated to either the research or underlying disease, disorder, or condition of the subject are not considered related or possibly related.

***Greater risk of harm than was previously known or recognized: Any event that is determined as 'serious' (see definition above) or an event that is not serious, but that warrants consideration of substantive changes in the research protocol or informed consent process/document or other corrective actions in order to protect the safety, welfare or rights of subjects.

Reporting of Adverse Events, Serious Adverse Events, and Unanticipated Problems:
Nonserious and serious adverse events and unanticipated problems will be reported to all appropriate regulatory bodies as required by current regulations.

***Reporting of non-serious adverse events**:* Upon notification of a non-serious AE, the Study Team member notified will notify all other team members and the following procedures will be followed:

1. Study staff will complete the AE form.
2. The PI will advise the Study Team regarding screening, enrollment, and ongoing participation and initiatives to prevent further AEs if relevant. The PI will evaluate the adverse event and determine whether the adverse event affects the risk/benefit ratio of the study and whether modifications to the protocol or consent form are required. The PI will be responsible for determining if the study protocol may be continued, terminated, or modified based on the observed beneficial or adverse effects of the treatment under study.
3. The PI will inform NIAMS and the DSMB through 6-monthly DSM reports submitted to the Executive Secretary (NCR).
4. If the non-serious AE is unexpected AND possibly/probably related or definitely related to the study intervention, study staff will report the event to the IRB within 5 business days (7 calendar days).

***Reporting of serious adverse events**:* Upon notification of a SAE, the Study Team member notified will immediately notify all other team members, in particular the study PI. The following procedures will be followed:

1. Study staff will complete the SAE form.
2. Study staff will notify NIAMS and the DSMB via the Executive Secretary (NCR) within 48 hours of learning of such events. This will be followed by a written report as soon as possible.
3. Study staff will notify the IRB within 5 business days (7 calendar days).
4. The PI will advise the Study Team regarding screening, enrollment, and ongoing participation.
5. Upon advisement by the IRB and DSMB, the PI will determine the study's status and notify the Study Team. A determination will also be made on whether modifications to the protocol and/or informed consent form/s are required.

Reporting of unanticipated problems: Upon notification of an unanticipated problem, the Study Team member notified will notify all other team members. The following procedures will be followed:

1. Study staff will complete the UP form.
2. Study staff will notify NIAMS and the DSMB via the Executive Secretary (NCR) within 48 hours of learning of such events. This will be followed by a written report as soon as possible.
3. Study staff will notify the IRB within 5 business days (7 calendar days). For UPs that are not adverse events, this will be reported to the IRB as an 'Other Event' via Insight.
4. The PI will advise the Study Team regarding screening, enrollment, and ongoing participation.
5. Upon advisement by the IRB and DMSB, the Principal Investigator will determine the study's status and notify the Study Team. A determination will also be made on whether modifications to the protocol and/or informed consent form/s are required.

Reporting to the FDA: Reporting to the FDA for non-IND studies is voluntary. The PI will adjudicate all serious adverse events that are suspected to be related or related to the use of the study drug and all medical product use errors in determining whether reporting to the FDA is necessary. In instances where the PI determines that an event should be reported, Form FDA

3500 will be used, and the report will be submitted within 14 calendar days of study staff becoming aware of the event. In the case that such an event causes death or is life-threatening, this will be reported to the FDA within 7 calendar days.

Time Period and Frequency for Event Assessment and Follow-Up
The occurrence of an AE or SAE may come to the attention of study personnel during study visits and interviews of a study participant presenting for medical care, or upon review by a study monitor. At each study visit, the investigator will inquire about the occurrence of AE/SAEs since the last visit.

All AEs including local and systemic reactions will be captured on the appropriate case report form. Information to be collected includes event description, time of onset, clinician's assessment of severity, relationship to study product (assessed only by those with the training and authority to make a diagnosis), and time of resolution/stabilization of the event. All AEs occurring while on study must be documented appropriately regardless of relationship.

Any medical condition that is present at the time that the participant is screened will be considered as baseline and not reported as an AE. However, if the study participant's condition deteriorates at any time during the study, it will be recorded as an AE.

Changes in the severity or seriousness of an AE will be documented to allow an assessment of the duration of the event at each level of severity and seriousness to be performed. AEs characterized as intermittent require documentation of onset and duration of each episode.

A Study Team member will record all reportable events with start dates occurring any time after informed consent is obtained until 7 days (for non-serious AEs) or 30 days (for SAEs) after the last day of study participation unless reporting is deemed necessary in the opinion of the study investigator. Events will be followed for outcome information until resolution or stabilization.

Interim Analysis: None planned

Stopping Rules: This study will be stopped prior to its completion if:
• The intervention is associated with adverse effects that call into question the safety of the intervention.
• Difficulty in study recruitment or retention impacts the ability to evaluate the study endpoints.
• New information becomes available during the trial that necessitates stopping the trial.
• Any situations that, in the opinion of the PI or DSMB, warrants stopping the trial.

Database Protection: The study database will be secured with password protection and only IRB-approved study staff will have access. The study biostatistician will receive only de-identified coded information. Electronic or written communication with outside collaborators will involve only unidentifiable information. Adverse event reports and annual continuing reviews will not include subject- or group-identifiable material. Data will be entered into REDCap, a free, secure, web-based application designed to support data capture for research studies. REDCap was developed by a multi-institutional consortium initiated at Vanderbilt University. Data collection will be customized for this study by the research team with guidance from Harvard Catalyst EDC Support Staff. REDCap is designed to comply with HIPAA regulations.

Monitoring of Source DATA: The PI and study team will meet at least once a month to review study activity. The activity includes recruitment, status of enrolled subjects, safety issues,

internal quality assurance and peer review information. Meeting minutes will be maintained documenting the date of the meeting, names of those in attendance, and summary of information discussed and reviewed. Research assistants/coordinators in the Endocrine Unit will exchange 1-2 subject files with each other on a quarterly basis as a supporting peer review process. Peer review will be completed in 1 week and include: review of informed consents, review of case report forms for accuracy and completeness, review of eCRFs for accuracy and completeness using source data verification. This process will be documented on the Peer Review Checklist. Additionally, independent monitoring of the source data will be at the discretion of the NAIMS-appointed DSMB as well as the MGH Human Research Quality Improvement (QI) program.

11. Privacy and Confidentiality

- ☒ Study procedures will be conducted in a private setting
- ☒ Only data and/or specimens necessary for the conduct of the study will be collected
- ☒ Data collected (paper and/or electronic) will be maintained in a secure location with appropriate protections such as password protection, encryption, physical security measures (locked files/areas)
- ☒ Specimens collected will be maintained in a secure location with appropriate protections (e.g. locked storage spaces, laboratory areas)
- ☒ Data and specimens will only be shared with individuals who are members of the IRB-approved research team or approved for sharing as described in this IRB protocol
- ☒ Data and/or specimens requiring transportation from one location or electronic space to another will be transported only in a secure manner (e.g. encrypted files, password protection, using chain-of-custody procedures, etc.)
- ☒ All electronic communication with participants will comply with Mass General Brigham secure communication policies
- ☒ Identifiers will be coded or removed as soon as feasible and access to files linking identifiers with coded data or specimens will be limited to the minimal necessary members of the research team required to conduct the research
- ☒ All staff are trained on and will follow the Mass General Brigham policies and procedures for maintaining appropriate confidentiality of research data and specimens
- ☒ The PI will ensure that all staff implement and follow any Research Information Service Office (RISO) requirements for this research
- ☐ Additional privacy and/or confidentiality protections

12. Protocol Amendment History

Version	Date	Description of Change	Brief Rationale
2.0	15 March 2022	Changes made in response to initial review of study protocol by NIAMS and the DSMB on 11/17/2021. The third inclusion criterion (3c) has been removed as per the NIAMS and DSMB recommendation on 03/10/2022.	Changes made in response to initial review of study protocol by NIAMS and the DSMB on 11/17/2021. Third inclusion criterion (3c) has been removed to improve applicability of study results.
3.0	22 July 2022	Prior therapy exclusion criteria changed from any current or previous use of teriparatide, abaloparatide, or any parenteral	Changes to prior therapy criteria made to aid in participant recruitment. Changes to PTH range made to align with Quest reference

		bisphosphonate to excluding just current use or use within the past 3 years of these medications. Exclusion criteria for elevated blood PTH changed from 65 to 77 pg/mL. Definition of major and minor protocol deviation added. Names of DSMB members updated in Appendix A. Language added to clarify that about 250 subjects will enroll in the screening part of the study and 46 subjects in the main part of the study.	range. Definitions of major and minor deviations added in response to DSMB recommendation 6/3/2022. Appendix A updated to reflect changes in DSMB membership. Clarified number of participants enrolled to reflect changes made to IRB study population forms during CR1 as requested by IRB.
4.0	22 August 2022	Change made to compensate subjects $20 per injection visit.	Change made to compensate subjects accordingly for their time.

13. References

1. Cummings SR, and Melton LJ. Epidemiology and outcomes of osteoporotic fractures. *Lancet.* 2002;359(9319):1761-7.
2. Looker A, Borrud LG, Dawson-Hughes B, Shepherd JA, and Wright NC. NCHS Data Brief: Osteoporosis or Low Bone Mass at the Femur Neck or Lumbar Spine in Older Adults: United States, 2005–2008. *National Center for Health Statistics, Centers for Disease Control and Prevention.* 2012;93(1).
3. Papapoulos SE. Use of bisphosphonates in the management of postmenopausal osteoporosis. *Ann N Y Acad Sci.* 2011;1218(1):15-32.
4. Cosman F. Parathyroid hormone treatment for osteoporosis. *Curr Opin Endocrinol Diabetes Obes.* 2008;15(6):495-501.
5. MacLean C, Newberry S, Maglione M, McMahon M, Ranganath V, Suttorp M, et al. Systematic review: comparative effectiveness of treatments to prevent fractures in men and women with low bone density or osteoporosis. *Ann Intern Med.* 2008;148(3):197-213.
6. Dempster DW, Zhou H, Recker RR, Brown JP, Bolognese MA, Recknor CP, et al. Skeletal histomorphometry in subjects on teriparatide or zoledronic acid therapy (SHOTZ) study: a randomized controlled trial. *J Clin Endocrinol Metab.* 2012;97(8):2799-808.
7. Ma YL, Marin F, Stepan J, Ish-Shalom S, Moricke R, Hawkins F, et al. Comparative effects of teriparatide and strontium ranelate in the periosteum of iliac crest biopsies in postmenopausal women with osteoporosis. *Bone.* 2011;48(5):972-8.
8. Stepan JJ, Burr DB, Li J, Ma YL, Petto H, Sipos A, et al. Histomorphometric changes by teriparatide in alendronate-pretreated women with osteoporosis. *Osteoporos Int.* 2010;21(12):2027-36.
9. Dobnig H, Stepan JJ, Burr DB, Li J, Michalska D, Sipos A, et al. Teriparatide reduces bone microdamage accumulation in postmenopausal women previously treated with alendronate. *J Bone Miner Res.* 2009;24(12):1998-2006.
10. Recker RR, Marin F, Ish-Shalom S, Moricke R, Hawkins F, Kapetanos G, et al. Comparative effects of teriparatide and strontium ranelate on bone biopsies and biochemical markers of bone turnover in postmenopausal women with osteoporosis. *J Bone Miner Res.* 2009;24(8):1358-68.
11. Jobke B, Pfeifer M, and Minne HW. Teriparatide following bisphosphonates: initial and long-term effects on microarchitecture and bone remodeling at the human iliac crest. *Connect Tissue Res.* 2009;50(1):46-54.
12. Neer RM, Arnaud CD, Zanchetta JR, Prince R, Gaich GA, Reginster JY, et al. Effect of parathyroid hormone (1-34) on fractures and bone mineral density in postmenopausal women with osteoporosis. *N Engl J Med.* 2001;344(19):1434-41.
13. Chen P, Satterwhite JH, Licata AA, Lewiecki EM, Sipos AA, Misurski DM, et al. Early changes in biochemical markers of bone formation predict BMD response to teriparatide in postmenopausal women with osteoporosis. *J Bone Miner Res.* 2005;20(6):962-70.
14. Ma YL, Zeng Q, Donley DW, Ste-Marie LG, Gallagher JC, Dalsky GP, et al. Teriparatide increases bone formation in modeling and remodeling osteons and enhances IGF-II immunoreactivity in postmenopausal women with osteoporosis. *J Bone Miner Res.* 2006;21(6):855-64.
15. Keaveny TM, McClung MR, Wan X, Kopperdahl DL, Mitlak BH, and Krohn K. Femoral strength in osteoporotic women treated with teriparatide or alendronate. *Bone.* 2012;50(1):165-70.

16. Fox J, Miller MA, Newman MK, Recker RR, Turner CH, and Smith SY. Effects of daily treatment with parathyroid hormone 1-84 for 16 months on density, architecture and biomechanical properties of cortical bone in adult ovariectomized rhesus monkeys. *Bone.* 2007;41(3):321-30.
17. Cosman F, Crittenden DB, Adachi JD, Binkley N, Czerwinski E, Ferrari S, et al. Romosozumab Treatment in Postmenopausal Women with Osteoporosis. *N Engl J Med.* 2016;375(16):1532-43.
18. McClung MR, Grauer A, Boonen S, Bolognese MA, Brown JP, Diez-Perez A, et al. Romosozumab in postmenopausal women with low bone mineral density. *N Engl J Med.* 2014;370(5):412-20.
19. Padhi D, Jang G, Stouch B, Fang L, and Posvar E. Single-dose, placebo-controlled, randomized study of AMG 785, a sclerostin monoclonal antibody. *J Bone Miner Res.* 2011;26(1):19-26.
20. Genant HK, Engelke K, Bolognese MA, Mautalen C, Brown JP, Recknor C, et al. Effects of Romosozumab Compared With Teriparatide on Bone Density and Mass at the Spine and Hip in Postmenopausal Women With Low Bone Mass. *J Bone Miner Res.* 2017;32(1):181-7.
21. McClung MR, Brown JP, Diez-Perez A, Resch H, Caminis J, Meisner P, et al. Effects of 24 Months of Treatment With Romosozumab Followed by 12 Months of Denosumab or Placebo in Postmenopausal Women With Low Bone Mineral Density: A Randomized, Double-Blind, Phase 2, Parallel Group Study. *J Bone Miner Res.* 2018.
22. Saag KG, Petersen J, Brandi ML, Karaplis AC, Lorentzon M, Thomas T, et al. Romosozumab or Alendronate for Fracture Prevention in Women with Osteoporosis. *N Engl J Med.* 2017.
23. Langdahl BL, Libanati C, Crittenden DB, Bolognese MA, Brown JP, Daizadeh NS, et al. Romosozumab (sclerostin monoclonal antibody) versus teriparatide in postmenopausal women with osteoporosis transitioning from oral bisphosphonate therapy: a randomised, open-label, phase 3 trial. *Lancet.* 2017;390(10102):1585-94.
24. Glover SJ, Eastell R, McCloskey EV, Rogers A, Garnero P, Lowery J, et al. Rapid and robust response of biochemical markers of bone formation to teriparatide therapy. *Bone.* 2009;45(6):1053-8.
25. McClung MR, San Martin J, Miller PD, Civitelli R, Bandeira F, Omizo M, et al. Opposite bone remodeling effects of teriparatide and alendronate in increasing bone mass. *Arch Intern Med.* 2005;165(15):1762-8.
26. Hansen S, Hauge EM, Jensen JE, and Brixen K. Differing effects of PTH 1-34, PTH 1-84 and zoledronic acid on bone microarchitecture and estimated strength in postmenopausal women with osteoporosis. An 18 month open-labeled observational study using HR-pQCT. *J Bone Miner Res.* 2012.
27. Macdonald HM, Nishiyama KK, Hanley DA, and Boyd SK. Changes in trabecular and cortical bone microarchitecture at peripheral sites associated with 18 months of teriparatide therapy in postmenopausal women with osteoporosis. *Osteoporos Int.* 2011;22(1):357-62.
28. Tsai JN, Uihlein AV, Burnett-Bowie SM, Neer RM, Derrico NP, Lee H, et al. Effects of Two Years of Teriparatide, Denosumab, or Both on Bone Microarchitecture and Strength (DATA-HRpQCT study). *J Clin Endocrinol Metab.* 2016:jc20161160.
29. Gardella T. In: Bilezikian J ed. *The Parathyroids*. New York, NY: Elsevier; 2015:65-80.
30. Leder BZ. Optimizing Sequential and Combined Anabolic and Antiresorptive Osteoporosis Therapy. *JBMR Plus.* 2018;2(2):62-8.

31. Finkelstein JS, Wyland JJ, Lee H, and Neer RM. Effects of teriparatide, alendronate, or both in women with postmenopausal osteoporosis. *J Clin Endocrinol Metab.* 2010;95(4):1838-45.
32. Leder BZ, Neer RM, Wyland JJ, Lee HW, Burnett-Bowie SM, and Finkelstein JS. Effects of teriparatide treatment and discontinuation in postmenopausal women and eugonadal men with osteoporosis. *J Clin Endocrinol Metab.* 2009;94(8):2915-21.
33. Prince R, Sipos A, Hossain A, Syversen U, Ish-Shalom S, Marcinowska E, et al. Sustained nonvertebral fragility fracture risk reduction after discontinuation of teriparatide treatment. *J Bone Miner Res.* 2005;20(9):1507-13.
34. Rittmaster RS, Bolognese M, Ettinger MP, Hanley DA, Hodsman AB, Kendler DL, et al. Enhancement of bone mass in osteoporotic women with parathyroid hormone followed by alendronate. *J Clin Endocrinol Metab.* 2000;85(6):2129-34.
35. Kurland ES, Heller SL, Diamond B, McMahon DJ, Cosman F, and Bilezikian JP. The importance of bisphosphonate therapy in maintaining bone mass in men after therapy with teriparatide [human parathyroid hormone(1-34)]. *Osteoporos Int.* 2004;15(12):992-7.
36. Black DM, Bilezikian JP, Ensrud KE, Greenspan SL, Palermo L, Hue T, et al. One year of alendronate after one year of parathyroid hormone (1-84) for osteoporosis. *N Engl J Med.* 2005;353(6):555-65.
37. Eastell R, Nickelsen T, Marin F, Barker C, Hadji P, Farrerons J, et al. Sequential treatment of severe postmenopausal osteoporosis after teriparatide: final results of the randomized, controlled European Study of Forsteo (EUROFORS). *J Bone Miner Res.* 2009;24(4):726-36.
38. Leder BZ, Tsai JN, Uihlein AV, Wallace PM, Lee H, Neer RM, et al. Denosumab and teriparatide transitions in postmenopausal osteoporosis (the DATA-Switch study): extension of a randomised controlled trial. *Lancet.* 2015;386(9999):1147-55.
39. Boonen S, Marin F, Obermayer-Pietsch B, Simoes ME, Barker C, Glass EV, et al. Effects of previous antiresorptive therapy on the bone mineral density response to two years of teriparatide treatment in postmenopausal women with osteoporosis. *J Clin Endocrinol Metab.* 2008;93(3):852-60.
40. Miller PD, Delmas PD, Lindsay R, Watts NB, Luckey M, Adachi J, et al. Early responsiveness of women with osteoporosis to teriparatide after therapy with alendronate or risedronate. *J Clin Endocrinol Metab.* 2008;93(10):3785-93.
41. Cosman F, Wermers RA, Recknor C, Mauck KF, Xie L, Glass EV, et al. Effects of teriparatide in postmenopausal women with osteoporosis on prior alendronate or raloxifene: differences between stopping and continuing the antiresorptive agent. *J Clin Endocrinol Metab.* 2009;94(10):3772-80.
42. Ettinger B, San Martin J, Crans G, and Pavo I. Differential effects of teriparatide on BMD after treatment with raloxifene or alendronate. *J Bone Miner Res.* 2004;19(5):745-51.
43. Cosman F, Nieves JW, Zion M, Garrett P, Neubort S, Dempster D, et al. Daily or Cyclical Teriparatide Treatment in Women With Osteoporosis on no Prior Therapy and Women on Alendronate. *J Clin Endocrinol Metab.* 2015;100(7):2769-76.
44. Compton JT, and Lee FY. A review of osteocyte function and the emerging importance of sclerostin. *J Bone Joint Surg Am.* 2014;96(19):1659-68.
45. Gardner JC, van Bezooijen RL, Mervis B, Hamdy NA, Lowik CW, Hamersma H, et al. Bone mineral density in sclerosteosis; affected individuals and gene carriers. *J Clin Endocrinol Metab.* 2005;90(12):6392-5.
46. Loots GG, Kneissel M, Keller H, Baptist M, Chang J, Collette NM, et al. Genomic deletion of a long-range bone enhancer misregulates sclerostin in Van Buchem disease. *Genome Res.* 2005;15(7):928-35.

47. Balemans W, Ebeling M, Patel N, Van Hul E, Olson P, Dioszegi M, et al. Increased bone density in sclerosteosis is due to the deficiency of a novel secreted protein (SOST). *Hum Mol Genet.* 2001;10(5):537-43.
48. Balemans W, Patel N, Ebeling M, Van Hul E, Wuyts W, Lacza C, et al. Identification of a 52 kb deletion downstream of the SOST gene in patients with van Buchem disease. *J Med Genet.* 2002;39(2):91-7.
49. Brunkow ME, Gardner JC, Van Ness J, Paeper BW, Kovacevich BR, Proll S, et al. Bone dysplasia sclerosteosis results from loss of the SOST gene product, a novel cystine knot-containing protein. *Am J Hum Genet.* 2001;68(3):577-89.
50. Staehling-Hampton K, Proll S, Paeper BW, Zhao L, Charmley P, Brown A, et al. A 52-kb deletion in the SOST-MEOX1 intergenic region on 17q12-q21 is associated with van Buchem disease in the Dutch population. *Am J Med Genet.* 2002;110(2):144-52.
51. Kogawa M, Wijenayaka AR, Ormsby RT, Thomas GP, Anderson PH, Bonewald LF, et al. Sclerostin regulates release of bone mineral by osteocytes by induction of carbonic anhydrase 2. *J Bone Miner Res.* 2013;28(12):2436-48.
52. Wijenayaka AR, Kogawa M, Lim HP, Bonewald LF, Findlay DM, and Atkins GJ. Sclerostin stimulates osteocyte support of osteoclast activity by a RANKL-dependent pathway. *PLoS One.* 2011;6(10):e25900.
53. Chavassieux P, Chapurlat R, Portero-Muzy N, Roux JP, Garcia P, Brown JP, et al. Bone-Forming and Antiresorptive Effects of Romosozumab in Postmenopausal Women With Osteoporosis: Bone Histomorphometry and Microcomputed Tomography Analysis After 2 and 12 Months of Treatment. *J Bone Miner Res.* 2019;34(9):1597-608.
54. Silvestrini G, Ballanti P, Leopizzi M, Sebastiani M, Berni S, Di Vito M, et al. Effects of intermittent parathyroid hormone (PTH) administration on SOST mRNA and protein in rat bone. *J Mol Histol.* 2007;38(4):261-9.
55. Keller H, and Kneissel M. SOST is a target gene for PTH in bone. *Bone.* 2005;37(2):148-58.
56. Drake MT, Srinivasan B, Modder UI, Peterson JM, McCready LK, Riggs BL, et al. Effects of parathyroid hormone treatment on circulating sclerostin levels in postmenopausal women. *J Clin Endocrinol Metab.* 2010;95(11):5056-62.
57. Bellido T, Ali AA, Gubrij I, Plotkin LI, Fu Q, O'Brien CA, et al. Chronic elevation of parathyroid hormone in mice reduces expression of sclerostin by osteocytes: a novel mechanism for hormonal control of osteoblastogenesis. *Endocrinology.* 2005;146(11):4577-83.
58. Jilka RL. Molecular and cellular mechanisms of the anabolic effect of intermittent PTH. *Bone.* 2007;40(6):1434-46.
59. Kramer I, Loots GG, Studer A, Keller H, and Kneissel M. Parathyroid hormone (PTH)-induced bone gain is blunted in SOST overexpressing and deficient mice. *J Bone Miner Res.* 2010;25(2):178-89.
60. Robling AG, Kedlaya R, Ellis SN, Childress PJ, Bidwell JP, Bellido T, et al. Anabolic and catabolic regimens of human parathyroid hormone 1-34 elicit bone- and envelope-specific attenuation of skeletal effects in Sost-deficient mice. *Endocrinology.* 2011;152(8):2963-75.
61. McClung MR, Brown JP, Diez-Perez A, Resch H, Caminis J, Meisner P, et al. Effects of 24 Months of Treatment With Romosozumab Followed by 12 Months of Denosumab or Placebo in Postmenopausal Women With Low Bone Mineral Density: A Randomized, Double-Blind, Phase 2, Parallel Group Study. *J Bone Miner Res.* 2018;33(8):1397-406.

APPENDIX A

Data Monitoring Committee / Data and Safety Monitoring Board Appendix

A Data Monitoring Committee (DMC) or Data and Safety Monitoring Board (DSMB) will be convened for safety monitoring of this research study. The DSMB will be appointed and managed by the funding agency (NIAMS). The following characteristics describe the DMC/DSMB convened for this study:

☒ The DMC/DSMB is independent from the study team and study sponsor.

☒ A process has been implemented to ensure absence of conflicts of interest by DMC/DSMB members.

☒ The DMC/DSMB has the authority to intervene on study progress in the event of safety concerns, e.g., to suspend or terminate a study if new safety concerns have been identified or need to be investigated.

☒ Describe number and types of (i.e., qualifications of) members: Elizabeth J. Shane, MD, *Chairperson*; Anne R. Cappola, MD, ScM, *Safety Officer*; Alex Kaizer, PhD; Jeri Nieves, PhD; Jennifer B. McCormick, PhD, MPP.

☒ Describe planned frequency of meetings: Every 6 months with the first meeting held on January 7, 2022

☒ DMC/DSMB reports with no findings (i.e., “continue without modifications”) will be submitted to the IRB at the time of Continuing Review.

☒ DMC/DSMB reports with findings/modifications required will be submitted promptly (within 5 business days/7 calendar days of becoming aware) to the IRB as an Other Event.
